# Effect of the nanoencapsulated sour tea (*Hibiscus sabdariffa* L.) extract with carboxymethylcellulose on quality and shelf life of chicken nugget

**DOI:** 10.1002/fsn3.1656

**Published:** 2020-05-26

**Authors:** Sahba Bahrami Feridoni, Dariush Khademi Shurmasti

**Affiliations:** ^1^ Department of Agriculture Savadkooh Branch Islamic Azad University Savadkooh Iran

**Keywords:** chicken nugget, lipid oxidation, nanocapsulate, oil uptake, sour tea

## Abstract

In this study, the antioxidant properties of sour tea extract (*Hibiscus sabdariffa* L.) were investigated in both free and nanoencapsulated forms to increase the quality and shelf life of chicken nugget during a 9‐day refrigerated storage period. For this purpose, the extract was prepared using ultrasound and quantities of phenolic and anthocyanin compounds, and antioxidant properties (DPPH free radical scavenging, FRAP) of extract was examined. The results showed that the value of phenolic compounds and anthocyanin compounds were 626.57 mg/g gallic acid and 379.11 µg/ml, respectively, and the extract had high antioxidant activity. Maltodextrin‐whey protein concentrate was used for nanoencapsulation of the extract. Then, to investigate the effect of sour tea extract (free and nanoencapsulated forms) with carboxymethylcellulose (CMC) on quality and shelf life of chicken nugget, five treatments, including T1: control, T2: CMC, T3: CMC +1,000 ppm extract, T4: CMC +1,000 ppm nano extract; and T5: CMC +TBHQ, were prepared. First, physicochemical properties of nugget were measured. The results showed that CMC and sour tea extract reduced oil uptake, moisture content, frying percentage, frying efficiency, and soft texture of fried chicken nugget, and overall the best results were observed in CMC treatment with both extract and nano‐extract (*p* < .05). Then, peroxide value (PV), total volatile nitrogen base (TVB‐N), thiobarbituric acid (TBA), and sensory indexes were evaluated in treatments stored at refrigerator for 9 days. The results showed that sour tea extract has antioxidant properties and coating of extract increased its antioxidant properties as nugget containing 1,000 ppm the nanoencapsulated sour tea extract with CMC delayed oxidative spoilage and organoleptic changes of chicken nugget (*p* < .05). Therefore, it seems that the nanoencapsulated sour tea extract with CMC can be used as a natural preservative in meat and meat products.

## Graphical abstract

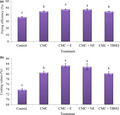

## 1 INTRODUCTION

Today, much of the food is produced industrially and marketed as ready to eat. These products have been able to gain a special position in the family and community food basket by meeting consumer needs such as acceptable sensory properties and high preparation speed. Ready to eat and semi ready to eat food such as fried products are a major part of this type of food (Alishahi, Ojagh, Shabanpour, & Izadi, 2017). Benefits of these foods include crisp texture, good appearance and color, quality improvement, and edible (Sanz, Salvador, & Fiszman, 2008). Chicken nuggets are a new product in the country that, due to their favorable taste, can increase the per capita consumption of meat products (chicken). Edible coatings are widely used in food industry due to their special sensory, chemical, and physical properties. Carboxymethylcellulose (CMC) is composed of two units, beta‐di‐glucose and beta‐di‐glucopyranose 2‐O‐carboxymethyl‐monosodium salt. It has the ability to form a good gel network. The use of CMC in food, especially due to its high biocompatibility, nontoxicity, biodegradation, flavor and etc. is increasing (Alishahi et al., 2017). However, the direct use of antibacterial substances on food limits its beneficial effects due to its neutralization or rapid release into the food. Therefore, methods have been developed to enrich edible coatings with antimicrobial and antioxidant substances to maintain high concentrations of these compounds in food to enhance the quality (Dehghan Nasiri, Mohebbi, Yazdi, & and. Khodaparast, 2012).

Addition of synthetic antioxidants such as butylated hydroxyanisole (BHA), butylated hydroxytoluene (BHT), and tert‐butyl hydroquinone (TBHQ) has widespread use as food additives in many countries. Recent reports reveal that these compounds may be implicated in many health risks, including cancer and carcinogenesis and it may cause liver swelling and influence liver enzyme activities. Much of the interest on naturally occurring antioxidants is developed because of the trend to minimize or avoid the use of synthetic food additives (Shahidi, 2005; Taghvaei & Jafari, 2015). Phenolic compounds present in plant extracts are responsible for the antimicrobial and antioxidants properties.

Sour tea (*Hibiscus sabdariffa L*.) belongs to the family Malvaceae. One‐year‐old plant, branched, about 69–429 cm high. The fruits are 2–5 cm long and are surrounded by sepals mainly for use as medicines. The sepals contain oxalic, malic, citricutartaric organic acids, proteins, minerals, and anthocyanins. Sepals are hypotensive, and due to their high vitamin C content, they have antioxidant properties (Ahmad, Shahlaby, & Shnan, 2011). Extraction of these bioactive compounds from the plant depends on several factors, the most important of which are solvents and extraction methods. One of the new methods of extraction is the use of ultrasound. This method is inexpensive, simple, and effective, and it is one of the most important advantages of high extraction efficiency and high reaction speed. In this method, a lower temperature is required for extraction, thus less damage to heat‐sensitive compounds. Compared to other extraction methods, this method is easier and cheaper and can be performed with different solvents (Maleki, Ariaii, & Fallah, 2016).

The bioactive compounds present in the extract and essential oil are volatile, some of which are insoluble in water and are easily oxidized. One method to overcome these limitations is the encapsulation of the extract. Encapsulation is a process in which fine particles and droplets of a material are coated with various materials to obtain useful properties. Some studies have shown that encapsulation methods are capable of enhancing the antimicrobial and antioxidant properties of the compounds as well as maintaining their properties for a longer time (Alipour, Javadian, & Bahram, 2016; Javadian, Shahoseini, & Ariaii, 2017).

The aim of the study was to extraction, nanoencapsulation (by maltodextrin and whey protein concentrate) of sour tea extract and effect of free and nano forms with CMC on the shelf life and quality properties of chicken nugget.

## 2 MATERIAL AND METHODS

### 2.1 Preparation of sour tea plant

The sour tea was purchased from the local market and was then packaged in impermeable plastic bags against light and moisture. The packages were stored at 4°C until extraction time. In order to extraction, the sour tea sepals were dried under vacuum oven (SH‐Scientific) at 50°C and 45 cm Hg for 45 min. It was completely powdered by crushing and stored at 25°C until the experiment.

### 2.2 Extraction by ultrasound method

The samples were first mixed with ethanol (80%) in a ratio of 1:5, then sonicated in an ultrasound bath (LUC‐410 model, Labtech) (total power consumption: 280 W, heating power: 200 W) for 45 min at 30°C with a frequency of 30–37 kHz. The solutions were filtered using Whatman No. 42 filter paper (Sigma‐Aldrich Chemical Co) and centrifuged for 10 min. Subsequently, the extract was concentrated by rotatory evaporator (maximum temperature 50°C). The extract was stored at 4°C until the experiment (Maleki et al., 2016).

### 2.3 Determination of total phenolic compounds

Folin–Ciocalteu test is one of the most common methods of phenolic compounds evaluation. The Folin reagent is reduced by the phenolic compounds in the alkaline medium and forms a blue color complex, which exhibits maximum absorption at 760 nm. Total phenolic compounds were determined according to the method described by Donald, Prenzler, Autolovich, and Robards (2001) based on Gallic acid.

### 2.4 Total anthocyanin measurement

Determination of total anthocyanin content by differential pH method was carried out according to method of Rapisarda, Fanella, and Maccarone (2005). One ml of clear solution of the extract was poured into two 25 ml volumetric flasks and then mixed with a buffer, separately and then both of them were filled with distilled water. In first volumetric flask, there is a buffer with pH 1(1.49 g potassium chloride with 100 ml water and 0.2N hydrochloric acid) and in another one there is a buffer with pH 5.4 (1.64 g sodium acetate with 100 ml). The absorbance of each of these solutions was measured by UV spectrophotometer at 510 nm and 700 nm, and the absorption difference of the diluted sample was calculated according to Eq. 1 (Rapisarda et al., 2005):

(1) $$\text{Ab}={\left(A510\text{nm}-A700\text{nm}\right)}_{\text{pH}=1}-\;{\left(A510\text{nm}-A700\text{nm}\right)}_{\text{pH}=5.4}$$

The total amount of anthocyanin in solution was calculated based Eq. 2:

(2) $$C\left(\text{mg}/100g\right)=\text{Ab}/e\times L\times \text{MW}\times \text{DF}\times V/G\times 100$$

e: 26,900 (The molar absorption of anthocyanin)L: 2.499 (The length of the pathway in cell)DF: Dilution factorV: The sample volume.MW: The molecular weightG: The sample weight in mg

### 2.5 DPPH free radical scavenging assay

This test was performed according to the method described by Maleki et al. (2016). DPPH (2, 20‐diphenyl‐1‐picrylhydrazyl) is a purple compound that is easily radicalized due to the presence of phenyl groups in its structure. An amount of 100 µl of each samples was mixed with 3 ml of DPPH in methanol (0.1 mM). After 30 min of incubation in the dark and in ambient temperature, absorbance was measured at 517 nm. The percentage scavenging was calculated according to the following equation:

$$\text{\textbackslash{}\% scavenging =}\;\frac{A_{\text{Contr}}{\;-\;A}_{\text{extr}}}{A_{\text{Contr}}}$$

where A_contr_ is the absorbance of the control (without extract) after 30 min and A_extr_ is the absorbance of extract after 30 min.

TBHQ (2‐(1,1‐dimethylethyl)‐1,4‐benzenediol) methanolic solutions were used as standards.

### 2.6 Ferric reducing antioxidant power assay (FRAP)

This test was performed according to the method of Benzie and Strain (1999). In this method, antioxidants have a reductive role and cause the reduction of Fe^+3^ to Fe^+2^ II. Depending on the regenerative power of the extract, the yellow color of the test solution changes to green or blue.

### 2.7 Preparation of the nanoencapsulated sour tea extract

Maltodextrin and whey protein concentrate were selected as coating for preparation of the nanoencapsulated sour tea extract. Nanoencapsulation was performed by Sharifi, Niakousari, Maskooki, and Mortazavi (2015) method. The maltodextrin and whey protein concentrate were dissolved in chloroform/methanol solution (1:3 w: w), then placed in a rotary evaporator to remove the solvents to form a thin film on the wall. The sour tea extract was also dissolved in dichloromethane/methanol solution (1:2 w: w) and the resulting mixture was combined with a 4:1 ratio of maltodextrin and whey protein concentrate (maltodextrin‐whey protein concentrate: extract) and finally solvents were evaporated under nitrogen vapor. The produced film was dissolved in 2 ml of phosphate buffer (10 mmol/L, pH 7.4). It was homogenized for 15 min at 35°C by a homogenizer at 200 bar pressure. The obtained suspension was incubated in the dark at room temperature for 2 hr. It was then centrifuged at 6,500 rpm at 4°C. Finally, the nanoencapsulated sour tea extract was dried using freeze dryer.

### 2.8 Nanoencapsulation efficiency

The encapsulation efficiency (%) of polyphenols was determined according to the method described by Jivan, Yarmand, and Madadlou (2014) using the following formula (Jivan et al., 2014):

$$100\times \text{EE}\%=\left(\text{Ce}/\text{Ct}\right)$$

where Ce is the content of polyphenols released from capsules (ppm), and Ct is the polyphenols content added into the particle formation solution (ppm). In this way, 0.6 g of powder with 20 ml of alkaline water (10.5 ppm) was mixed with a magnetic mixer and then stirred for 30 min. Then, it was centrifuged at 4,000 rpm for 10 min. The supernatant phase was reached to pH = 7.

### 2.9 Measurement of particle size

The mean diameter, particle size distribution, and particle specific area were measured using a laser light refraction device (Zetasizer nano zs. Malvern Co.). The mean diameter of the particles was represented by the symbol 43d (diameter of volume‐length) and was calculated according to the following equation. In this formula, zi will be the number of particles with di diameters (Joey, Davidov‐Pardo, & McClements, 2015).

$$D_{4,3}=\sum n_{i}d_{i}^{4}/\sum n_{i}d_{i}^{3}$$

### 2.10 Materials used in coating

Percentage of CMC examined (2%) in this study, a review of previous studies in this field was used. For primary flouring in coated flour from wheat flour with different coating percentages formula 1:100% wheat flour, formula 2:98% wheat flour and 2% CMC, formula 3:98% wheat flour and 2% CMC with sour tea free extract (percentage determined by antioxidant tests), formula 4:98% wheat flour and 2% CMC with the nanoencapsulated sour tea extract by maltodextrin and whey protein concentrate (WPC) (percentage determined by antioxidant assays), and formula 5:98% wheat flour and 2% CMC with synthetic antioxidant TBHQ (100 ppm) (Alishahi et al., 2017).

The glaze formula was prepared according to Chen, Chen, Chao, and Lin (2009) method which consisted of 55% wheat flour, 30% oxidized starch flour, 10% gluten flour, 2% baking powder, and 3% salt. In the final part of the coating, was a used orange breadcrumb with medium particle size.

### 2.11 Chicken nugget preparation

Approximately 30 fillets and seven drumsticks were freshly purchased from the local market and transported to the laboratory under the correct conditions. Fillets and drumsticks were washed and deboned and then were minced using a meat grinder with a 3 mm pore diameter.

According to the formulation of chicken nuggets in Table 1, the size of the chicken nuggets was round molded into dimensions of 5 cm in diameter and about 1 cm in height, then the primary flour was immersed in the glaze and after removing the excess glaze (after one minute), and they were coated with medium grain industrial breadcrumbs. After this, the nuggets are prefried with sunflower oil (fried) for 1 min at 180°C (standard method) and deep fried to maintain their product shape. After cooling to ambient temperature, the replicates of each treatment were packed into zip‐packs and frozen in −18°C. After frying each treatment, the oil was replaced. After three days, the chicken nuggets were defreezed and fried in a deep frying process for 2.5 min at 180°C for future experiments (Alishahi et al., 2017).

**Table 1:** Formulation and ingredients of chicken nuggets (control treatment)

Row	Components	%
1	Chicken meat (Thigh & Breast 4:1 ratio)	93.5
2	Salt	1.5
3	Sugar	1
4	Pepper	0.24
5	Black cumin	0.24
6	Onion powder	0.24
7	Garlic powder	0.24
8	Wheat flour	3
9	Thyme	0.2

In total, five treatments were examined:

1. Control
2. CMC (2%)
3. CMC (2%) + free of extract sour tea at 1,000 ppm
4. CMC (2%) + nanoencapsulated sour tea extract at 1,000 ppm
5. CMC (2%) + TBHQ at 100 ppm

After preparation of different treatments, physicochemical, texture, and sensory tests on the nuggets were performed. Also, the nuggets were transferred to the refrigerator and stored at 4°C for 9 days and were chemically evaluated at 0, 3, 6, and 9 days of intervals.

### 2.12 Physicochemical tests

#### Moisture content

Approximately 5–10 g of the nugget sample was placed in an oven at 105°C for 4 hr and transferred to a desiccator. Moisture content was calculated according to the following formula (AOAC, 2005).

$$\text{Moisture content}=\left(\text{final weight}-\;\text{Chinese cruise}/\text{initial weight}\right)\times 100$$

#### 2.12.1 Oil uptake value

Extraction and measurement of fat were performed according to standard method (AOAC, 2005) using soxhlet method. The dried chicken nugget was crushed completely, and the fat was soxhlet in an extractor using petroleum ether. The percentage of oil absorbed was obtained from the difference between the fat content of chicken nugget before and after frying.

#### 2.12.2 Texture analysis

To measure nugget's texture, the samples were cooked at 170°C for 5–10 min and then stored at 4°C for 12 hr to reduce the center temperature of the samples to 4°C. Then, the cubic samples were cut in 1 × 1 × 1 cm^3^ and subjected to compression test by a flat probe texture analyzer (25/000 TA) with dimensions of 20 × 50.8 mm and 10 kg load. The force required to compress the samples to 70% of their initial height at a constant speed of 1 mm/min (1) indicates texture firmness (Vural, 2003).

#### 2.12.3 Nuggets coating percentage

The percentage of coating is calculated by the following equation:

$$\text{CP}\left(\%\right)=C-I/I\times 100$$

In this equation, CP (%), C, and I are the percentage of coating, the weight of the initial coated slices (g), and the weight of the initial uncoated slices (g), respectively (Daraei Garmakhany, Mirzaei, Maghsudlo, Kashani Nejad, & Jafari, 2012).

#### 2.12.4 Frying efficiency

The frying efficiency is calculated by the following equation:

$$Y\left(\%\right)=F/\text{NF}\times 100$$

In this equation, Y (%), F and NF are frying efficiency (%), the weight of fried coated slices (control) (g), and weight of nonfried coated slices (g), respectively (Daraei Garmakhany et al., 2012).

#### 2.12.5 Oxidation indices

Peroxide value: The peroxide value (PV) measures the amount of primary oxidation products (hydroperoxides). Peroxide value of the samples was determined according to the Pearson method (Bagheri, Izadi Amoli, Tab ari Shahndash, & Shahosseini, 2016). Results were expressed in meq oxygen kg‐1 lipids.

Thiobarbituric acid: Thiobarbituric acid indicates secondary oxidation products (malondialdehyde). This test was performed according to Valipour Kootenaie, Ariaii, Khademi Shurmasti, and Nemati (2016) and expressed as mg malondialdehyde/kg sample.

The total volatile basic nitrogen (TVB‐N) of samples was determined by the micro‐diffusion method as described by Javadian et al. (2017). Results were expressed as mg N/100 g of samples.

#### 2.12.6 Sensory characterizations

Sensory properties of chicken nugget samples were evaluated by 10 semi‐trained assessors on color, odor, and general acceptance on the first and last day of storage by a five‐point hedonic test with a score of 5 indicating very good and a score of 1 indicating very bad (Suarez, Campanone, Garcıa, & Zaritzky, 2008). Each evaluator will be considered a block, and the data of sensory tests will be analyzed, as nonparametric, by completely randomized block design with SPSS software at 95% confidence level.

### 2.13 Statistical analysis

All experiments were performed in completely randomized design as triplicates, and the result was reported as mean ± *SD*. Statistical analysis of treatments was performed by ANOVA using SPSS software. Significant mean differences were determined by Duncan test at 0.05 level, and charts were plotted using Microsoft Excel software.

## 3 RESULTS AND DISCUSSION

Phenolic compounds in fruits and vegetables have attracted the attention of many researchers due to their high potential for antioxidant activity. Phenolic compounds by donating hydrogen atoms prevent free radical activity (Haji mahmmodi et al., 2012). The value of phenolic compounds was 626.57 ± 16.27 mg gallic acid/gr. Phenolic compounds of sour tea extract were higher than the values of phenolic compounds of sour tea reported by Formagio et al. (2015), their phenolic compounds content being 377.78 ± 6.61 mg/gallic acid/gr. This may be due to differences in extraction methods. Formagio et al. (2015) used from solvent method but ultrasound method was used in this study. In fact, ultrasound waves facilitate both the extraction process, that is, the swelling of the tissue as well as the exit of its constituents through the formation of pores and cavity in the cell wall and improved diffusion and mass transfer, which enhances the permeability of the solvent to the cell tissues. The mechanical effects of ultrasound arise, thereby destroying living cells as a result of these waves, releasing their materials better and easier (Kadam, Tiwari, Smyth, & Donnell, 2015).

Anthocyanins are heterocyclic compounds that can be chemically classified into both families of flavonoids and phenolics. Anthocyanins are widely distributed in nature and are among the best known natural pigments responsible for the development of blue, purple, red, and orange dyes. A characteristic that doubles the importance of anthocyanins is their medicinal properties. Medicinal and therapeutic properties of anthocyanins including antioxidant, anti‐inflammatory, antimicrobial, and anticancer which are at the forefront of their use in some cardiovascular diseases (Dent et al., 2015). In this study, the amount of anthocyanin compounds was 379.11 ± 10.88 µg/ml. Mak, Chuah, Ahmad, and Bhat (2013) reported the amounts of anthocyanin compounds in sour tea extract by alcoholic and aqueous solvent extraction methods between 155.28 – 205.76 μg/ml, which are lower than the present study.

### 3.1 DPPH free radical scavenging activity

**FIGURE 1:**
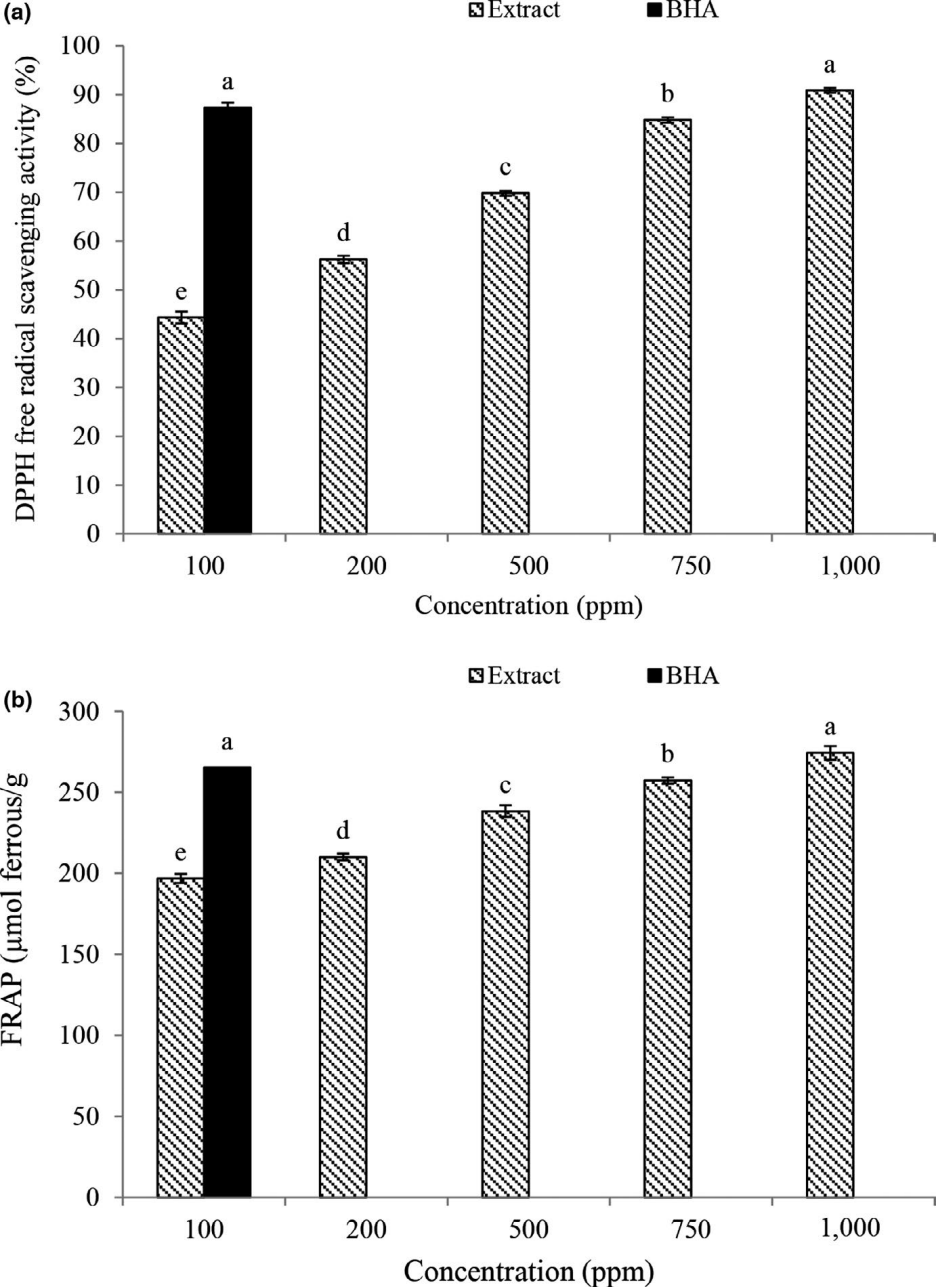
Effects of extract concentration and BHA on the antioxidant activity (DPPH (a) and FRAP (b)). Different small letters represent signiﬁcant difference (*p* < .05)

2‐Diphenyl 2‐Piperylhydrazyl (DPPH) is a stable radical, with a maximum absorption of about 515 nm, and can be rapidly inactivated with an antioxidant. This method has been widely used to measure the amount of free radical inhibition of various compounds (Hatamnia, Abbaspour, & Darvishzadeh, 2014). According to the results, DPPH radical scavenging (Figure 1a) was affected by different concentrations of the extract and increased with increasing concentration of extract (*p* < .05). The highest DPPH radical scavenging activity was observed at 1,000 ppm (87.86%). The concentration of antioxidant activity at this concentration was not significantly different from the synthetic antioxidant BHA (*p* < .05). Plant extracts have antioxidant activity and high capacity to donate hydrogen atoms or free electrons due to the presence of phenolic compounds, and by increasing the concentration of phenolic compounds or the degree of hydroxylation of phenolic compounds, the radical scavenging activity of the essential oil or extract is increased (Maleki et al., 2016). The antioxidant property of sour tea extract has also been reported in the study of Mak et al. (2013) and Formagio et al. (2015).

### 3.2 Ferric reducing antioxidant power assay (FRAP)

The presence of reducing agents in the extract reduces ferric ions (Fe ^+3^) to ferrous ions.

(Fe ^+2^). This reaction is measured by blue‐green at a wavelength of 700 nm (Do et al., 2014). The rate of reductive iron (FRAP) (Figure 1b) was affected by the concentration of extract and increased with increasing FRAP activity. The highest FRAP was observed at 1,000 ppm (89.17%). The concentration of antioxidant activity at this concentration was not significantly different from the synthetic antioxidant BHA. The antioxidant activity of the reducing agents in the extract is based on the breakdown of the free radical chain formation by electron donation or hydrogen atoms. Also during the extraction process, other compounds that are highly soluble in water and alcoholic solutions enter the extracts with phenolic compounds, and since some of these compounds, such as ascorbic acid, proteins, and sugars, are electron donors themselves and more percent of Fe ^+3^ ions by absorbing electrons, are reduced and thus the adsorption intensity is increased. Therefore, increasing the concentration of the extract increases all of the above compounds and, as a result, FRAP is increased (Sannigrahi, Mazuder, Pal, Parida, & Jain, 2010).

### 3.3 Nanoencapsulation tests

Particle size and particle size distribution are of particular importance in determining the properties of colloidal systems. The values and stability of these two parameters play an important role in determining the stability of the colloidal carrier system and its encapsulation efficiency. In this study, sour tea extract was nanoencapsulated by maltodextrin‐protein whey concentrate and the particle size of the nanoencapsulated extract in the present study was 139.03 ± 2.76 nm, and the nanoencapsulation efficiency was 67.30 ± 1.37%. According to the results, the nanoencapsulated extract is small in size. Smaller size of nano‐extract have greater stability due to higher resistance to gravity due to Brownian motion (Fathi, Mozafari, & Mohebbi, 2012). Also, the nanoencapsulated extract has high efficiency. Noshad, Mohebbi, Koocheki, and Shahidi (2015) reported the vanillin encapsulation efficiency of maltodextrin and soy proteins isolate 51.9%.

### 3.4 Moisture value and oil uptake in fried nuggets

**FIGURE 2:**
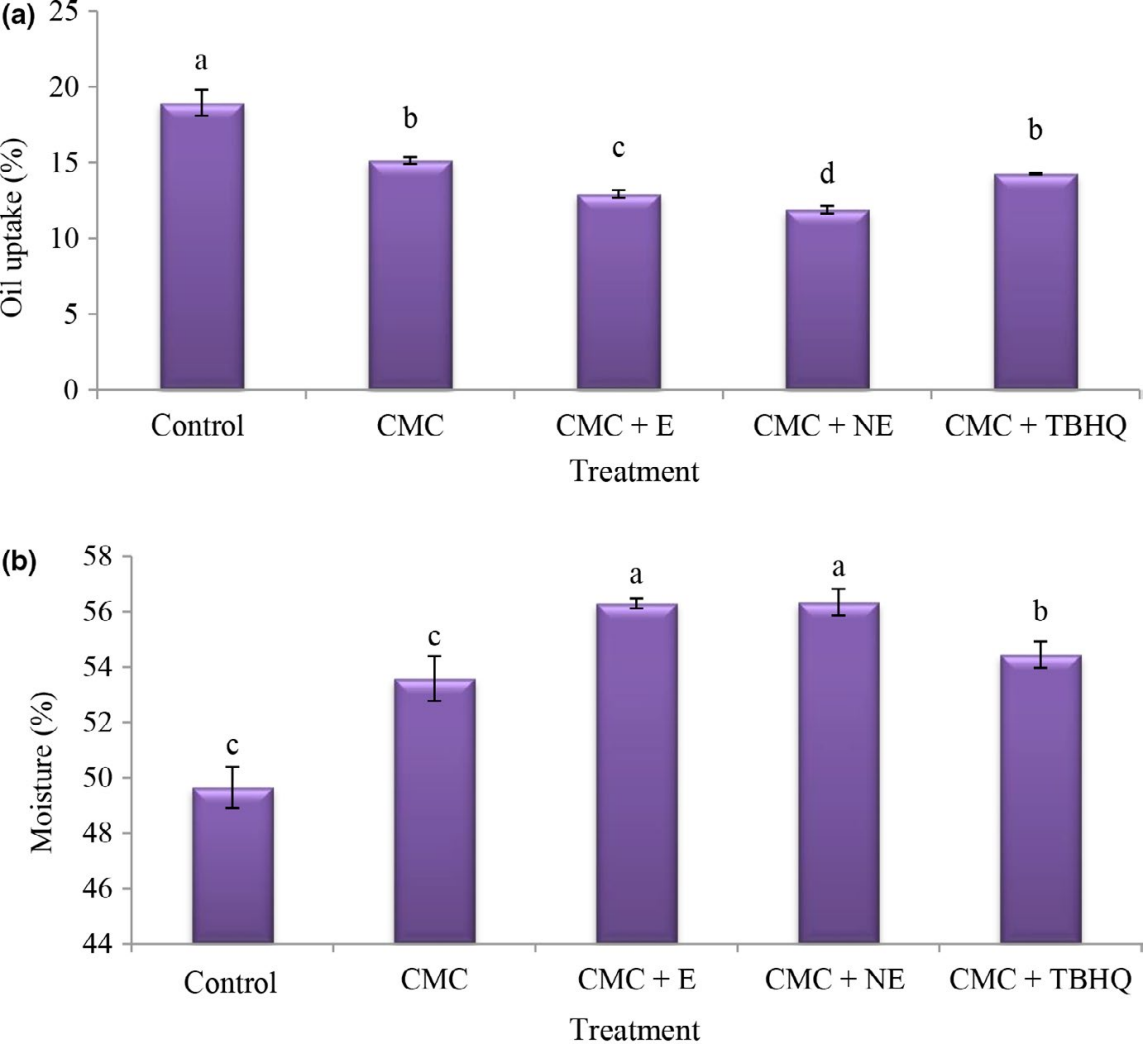
The amounts of oil uptake (a) and moisture (b) of different treatment. Different small letters represent signiﬁcant difference (*p* < .05)

Fried products have a high fat content due to the frying process and oil uptake. The surface properties of the food, the initial moisture content, the temperature and time of frying, and the degree of hydrogenation of the oil have an important effect on the absorption of fried products (Song, Liu, Shen, You, & Luo, 2012). According to the results, the highest oil uptake (Figure 2a) was observed in the control treatment (18/95) and the addition of CMC with the extract as well as the nanoencapsulated extract reduced the oil uptake. The lowest oil uptake was observed in CMC + nano‐extract (1,000 ppm). Bonding the extract with water molecules and preventing it from being replaced with oil as well as hydrocolloidal coatings by forming a gel network and maintaining this network during the frying process reduces oil absorption during frying and consequently reduces energy (Haghshenas, Hosseini, Nayebzadeh, Rashedi, & Rahmatzadeh, 2013).

According to the results, the lowest moisture (Figure 2b) content was observed in the control treatment and addition of CMC increased the moisture content and addition of the extract showed higher moisture content. The highest moisture content was observed in the CMC + extract and nano‐extract at 1,000 ppm. In general, moisture content increased and oil absorption decreased with increasing concentration of hydrocolloid solutions. This may be due to the combined effect of the gel forming properties during heating and the high degree of coating, creating a suitable and relatively thick layer against moisture outflow and oil penetration into the fillets. In the frying process, structural changes in food due to the high temperature of the oil decrease the moisture content. Reducing the moisture content of the product increases the porosity. During frying, it replaces the moisture lost in the small and large cavities. Overall, coatings can partially prevent oil entry and moisture outflow (Haghshenas et al., 2013). According to National Iranian Standard No. 9,868, the maximum moisture content in nugget and chicken burger was 58%. In this study, all treatments had standard moisture content.

### 3.5 Frying efficiency value in fried nugget

**FIGURE 3:**
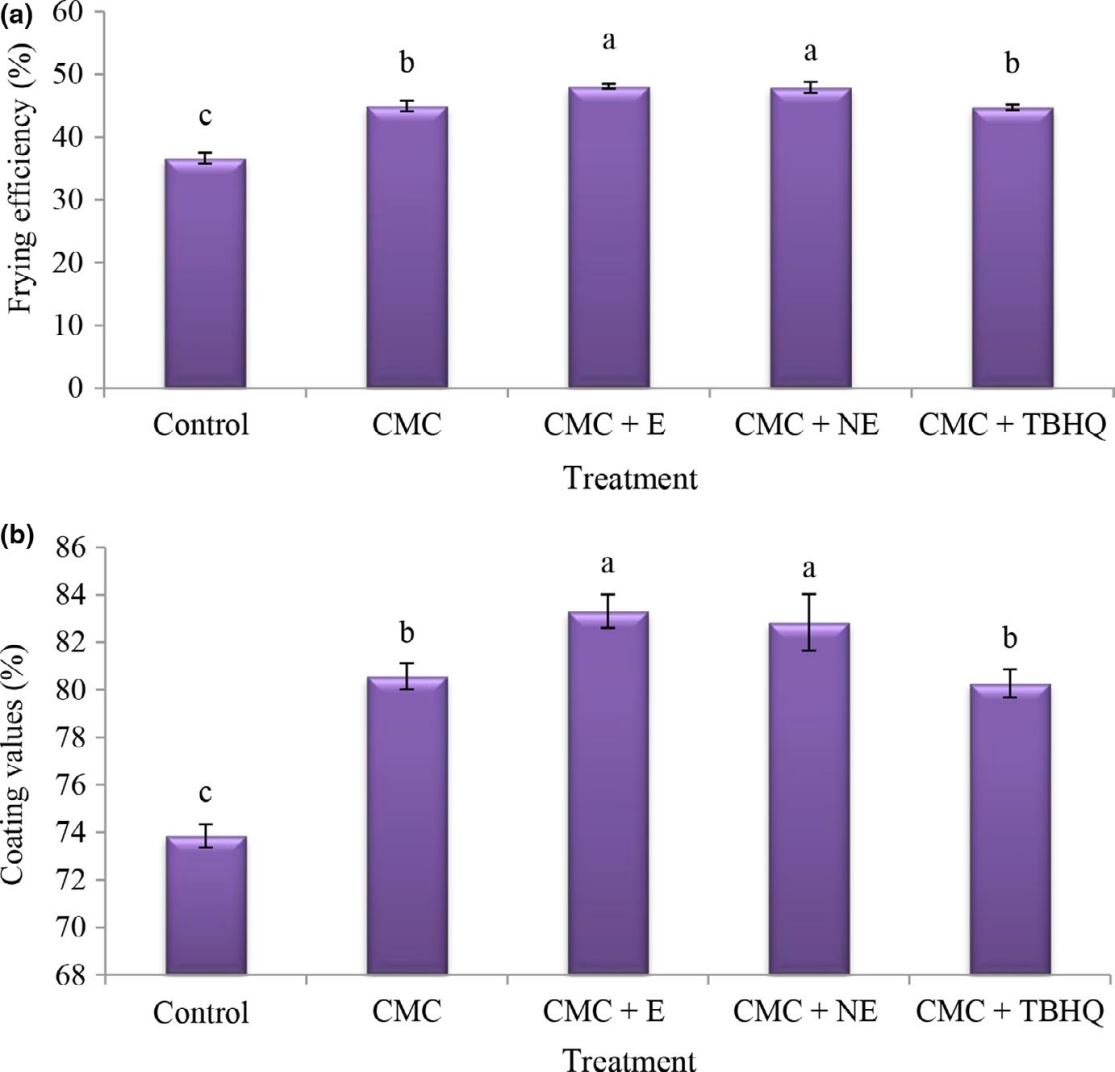
The amounts of frying efficiency (a) and coating value (b) of different treatment

The frying efficiency shows the sample ratio before and after frying. According to the results, the lowest values of frying efficiency (Figure 3a) were observed in the control treatment (36.66%), and the addition of CMC increased the frying efficiency, and the addition of extract significantly increased the frying efficiency. High uptake of oil during frying causes breakdown of cell tissue and frying efficiency is higher in the CMC + extract and nano‐extract 1,000 ppm due to lower oil uptake. Sakhale, Badgujar, Pawar, and Sananse (2011) reported that xanthan gum reduced oil uptake until 8.56% in a samosa, and they also stated that frying efficiency increased as the coating concentration increased due to the barrier effects of the coating.

### 3.6 Coating values in fried nugget

According to the results, the lowest amount of coating (Figure 3b) was observed in the control treatment and the addition of CMC increased the amount of coating and also by adding more extract, the highest amount of coating was observed in the treatment of CMC + nano‐extract and 1,000 ppm extract. Chen, Kang, and Chen (2008) stated that the viscosity of the hydrocolloid coatings plays an important role in the percentage of coatings; that is, with increasing hydrocolloids concentration, more coating is attached to the fish finger and increases the coating percentage.

### 3.7 Texture firmness values in fried nugget

**FIGURE 4:**
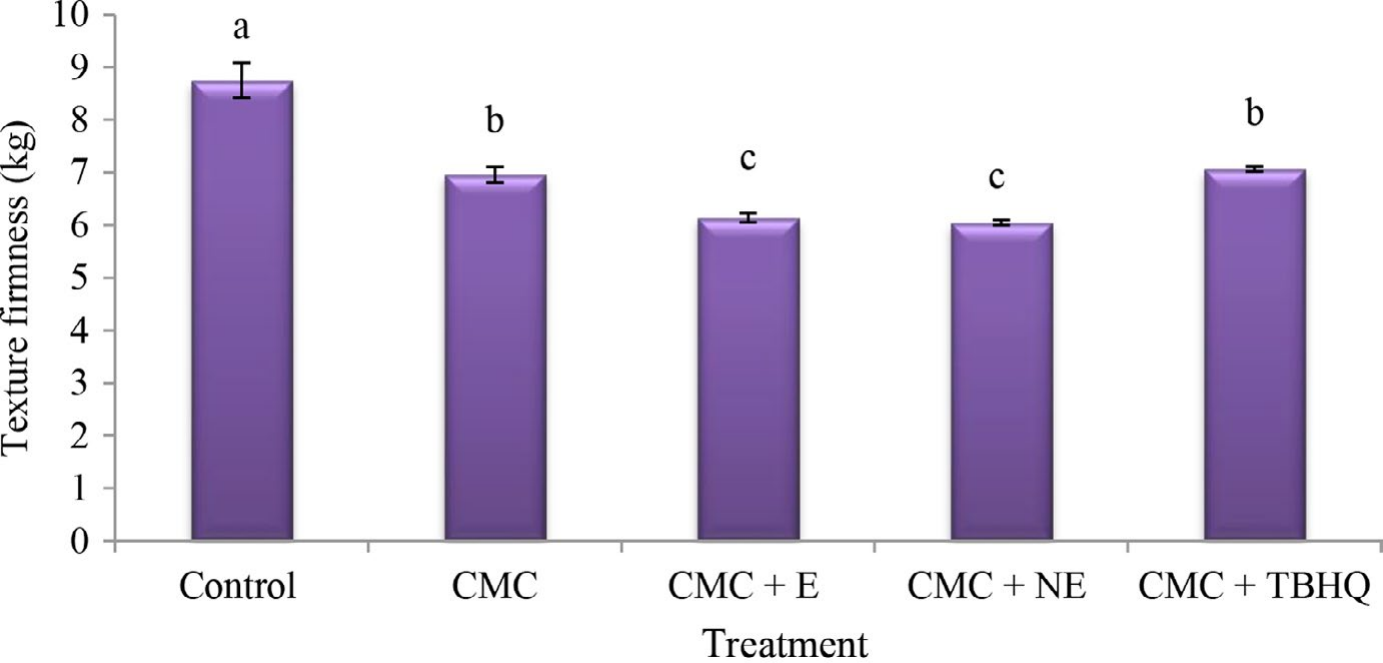
The amounts of texture firmness of different treatment

Firmness is the characteristic of a texture that defines the amount of force required to change the shape of a product and is defined as the force the consumer uses tooth to compress the product. The highest values of firmness (Figure 4) were observed in the control treatment, and the addition of CMC reduces the bonds between the meat proteins and reduces the firmness of the product (Alishahi et al., 2017). These results are in agreement with those of Polizer, (2015) regarding the addition of pea fiber to chicken nugget.

### 3.8 Peroxide value changes during storage

Oxidation of fat is one of the main causes of spoilage during storage that causes undesirable odor and taste as well as loss in nutritional value. The peroxide value is used to determine the formation of hydroperoxides (The primary compounds of oxidation). Therefore, determination of PV in meat samples is necessary for oxidation of meat fat (Valipour Kootenaie et al., 2016).

**FIGURE 5:**
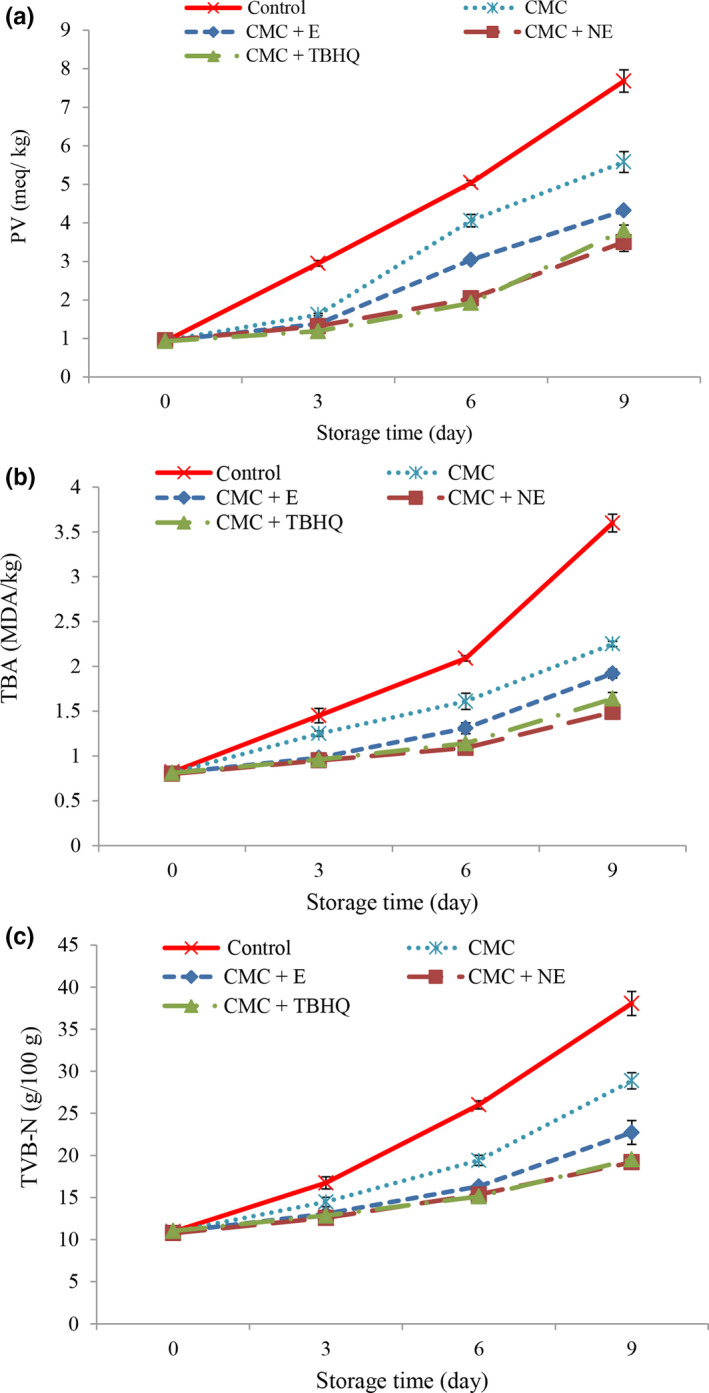
Changes in peroxide value (a), thiobarbituric acid (b), and total volatile base nitrogen (c) of different treatment during storage

The results of PV in chicken nuggets are presented in Figure 5a. Over time, PV increased significantly in all treatments (*p* < .05). But on other days of storage, the addition of natural preservatives (sour tea extract) slowed the increase in peroxide value. Hydrocolloids, such as CMC, prevent oxygen penetration into the tissue; thereby reduce the rate of initial oxidation of fats and subsequent formation of hydroperoxides. Phenolic compounds in sour tea extract also act as electron donors and may be neutralized the unwanted reactions created by free radicals in the body. In fact, polyphenols are capable of trapping free radicals, especially proxy radicals, which are one of the key intermediate chain reactants, thereby terminating the cycle of oxidative spoilage reactions and reducing the rate of increase of PV during storage (Bagheri et al., 2016; Valipour Kootenaie et al., 2016).

Overall, the peroxide value was lower in the nano‐coating treatments than in the other treatments. Encapsulation increases antioxidant activity, and nanoencapsulation protects hydrocolloids from environmental factors such as pH, oxygen, light, and so on. Volatile molecules also remain stable in this way, protecting them from oxidative, light, and volatile conditions. Therefore, nanoencapsulation enhance bioavailability, improve release control, and aim to precisely incorporate bio‐compounds as a result of improved antioxidant activity (Bagheri et al., 2016).

The acceptable level of peroxide value in meat products is five for human consumption (Yanar, 2007). Except for control and CMC, other treatments were within the standard range until the end of storage.

### 3.9 Thiobarbituric acid changes during storage

Thiobarbituric acid index is widely used to evaluate the degree of lipid oxidation in food and indicates the rate of secondary oxidation products, especially aldehydes and ketones. Secondary oxidation compounds cause unpleasant odors in the meat (Alipour et al., 2016).

Results for TBA values in chicken nugget are presented in Figure 5b. Over time, the amount of TBA increased significantly in all treatments (*p* < .05). During the storage period, the addition of natural preservatives (sour tea extract) slowed the increase in TBA (*p* < .05). Chen, Jo, Lee, and Ahn (1999) reported the possibility of use of dried and extract plants. They are found in meat products to reduce the oxidation of fats. The compounds present in the extracts are suitable donors of electrons and protons, and their intermediate radicals are very stable due to the phenomenon of electron movement in the benzene ring and the lack of an oxygen‐sensitive site. Compounds found in sour tea extract have the potential to neutralize free radicals and are also able to inhibit metal ions such as Fe^+2^, thereby reducing the rate of formation of reactive oxygen molecules (Mohamed & Mansour, 2012).

Also, the results were better in the treatments containing the nanoencapsulated extract so that on the 9th day, the lowest values of TBA were observed in the nanoencapsulated extract with 1,000 ppm (*p* < .05). In fact, it can be stated that the nanoencapsulation of sour tea extract increases its antioxidant activity and prolongs its effectiveness during storage. The results of this study are in agreement with the results of Alipour et al. (2016) regarding the addition of nanoencapsulated fennel extract to Silver carp fillets. They also reported that the use of the nanoencapsulated extract slowed down the change in the value of TBA during storage.

Generally, TBA levels of 2 mg malondialdehyde/g of meat are considered as acceptable of consumption (Campo et al., 2006). At the end of storage period, TBA levels in control and CMC treatments were not acceptable levels. However, other treatments were in permissible range.

### 3.10 Total volatile base nitrogen changes during storage

Total volatile nitrogen base is mainly produced by bacterial and enzymatic degradation of meat proteins and nonprotein nitrogen compounds. TVB‐N is a general term that includes trimethylamine (caused by bacterial spoilage), dimethylamine (produced by autolytic enzymes during storage), ammonia (caused by the decontamination of amino acids and nucleotide catabolites), and other volatile nitrogen compounds related to food spoilage (Valipour Kootenaie et al., 2016).

The results of the amounts of TVB‐N in chicken nugget are presented in Figure 5c. With increasing time, the amount of TVB‐N in all treatments increased significantly, and this was higher in control treatment. The increase in TVB‐*N* values in the samples can be attributed to the activity of spoilage bacteria, such as compounds such as trimethylamine oxide and peptides and amino acids being broken down by their high activity into volatile bases (López‐Caballero, Martínez‐Alvarez, Gómez‐Guillén, & Montero, 2007). Since the presence of bacteria in the meat leads to the autolysis of the proteins and their breakdown, breaking down of compounds such as trimethylamine oxides, peptides, and amino acids, the higher bacterial loads observed in the control samples can be justified for increasing the amount TVB‐N in them. During the storage period, the addition of synthetic and natural antioxidants reduced TVB‐N. The lower TVB‐N in this treatment than the other treatments can be due to the reduction of the bacterial population or the reduced ability of the bacteria to extract amines from nonvolatile nitrogen compounds or both due to the effect of sour tea extract on the bacteria in nuggets. The extract also has an antibacterial effect due to its phenolic compounds, as well as the presence of a protective layer (CMC) which acts as a barrier and results in lower protein quality than the control treatment. These properties are exacerbated when the CMC coating is combined with the nano‐extract (Valipour Kootenaie et al., 2016). The lowest values of TVB‐N were observed in the nanoencapsulated extract at 1,000 ppm (*p* < .05). This is due to the increased antibacterial activity of the coatings after encapsulation or the maintenance of antibacterial properties for a longer period after encapsulation (Javadian et al., 2017).

The desirable limit of TVB‐N in meat products has been reported to be 25 mg/100 g (Ashour, Moawad, & Bareh, 2013). At the end of storage period, TVB‐N levels control and CMC treatments were not acceptable levels. However, other treatments were in permissible range.

### 3.11 Sensory evaluation during storage

Undoubtedly, the sensory characteristics of chicken nuggets are one of the most important acceptance factors from a consumer perspective. Therefore, the evaluation of sensory characteristics is very important considering the market value of the product and also the sensory analysis is the ultimate guide to product acceptance by evaluators. Therefore, it is important to examine the sensory characteristics. The results of sensory analysis of different chicken nugget treatments including color, odor, taste, texture, and general acceptance are presented in Table 2. The results are as follows A: excellent, B: good, C: moderate, D: bad, and E: very bad.

**Table 2:** Sensory evaluation in different treatments during storage

Sensory attributes	Treatment	Storage time (day)	
		9	0
Color	Control	5.00 ± 0.00^a^	2.10 ± 0.73^a^
	CMC	4.90 ± 0.31^ab^	3.10 ± 0.75^b^
	CMC + extract	4.70 ± 0.48^ab^	4.00 ± 0.47^a^
	CMC + nano‐extract	4.80 ± 0.42^ab^	4.20 ± 0.42^a^
	CMC + TBHQ	4.60 ± 0.51^b^	4.30 ± 0.43^a^
Odor	Control	5.00 ± 0.00^a^	2.10 ± 0.73^a^
	CMC	5.00 ± 0.00^a^	3.30 ± 0.73^b^
	CMC + extract	5.00 ± 0.00^a^	4.10 ± 0.47^b^
	CMC + nano‐extract	5.00 ± 0.00^a^	4.00 ± 0.42^ab^
	CMC + TBHQ	5.00 ± 0.00^a^	4.30 ± 0.43^a^
Overall acceptance	Control	5.00 ± 0.00^a^	1.90 ± 0.87^a^
	CMC	4.80 ± 0.42^ab^	3.40 ± 0.69^b^
	CMC + extract	4.70 ± 0.48^ab ^	3.90 ± 0.56^ab^
	CMC + nano‐extract	4.80 ± 0.42^ab^	4.40 ± 0.69^a^
	CMC + TBHQ	4.80 ± 0.42^ab^	4.40 ± 0.84^a^

Note

a,b,c With different small letters in the same row, represents significant difference (*p* < .05).

The results did not change the odor and color of the nugget by adding preservatives. With regard to color and overall acceptance, sensory scores were significantly reduced by adding preservatives. But all treatments had sensory ratings approved by the evaluators.

At the end storage, color in the control treatment very bad and the best color was observed in the CMC + nano‐extract treatment. This treatment was not significantly different from CMC + TBHQ treatment. With longer storage time, the intensity of odor changes as well as the general acceptance was observed in all treatments but at the end of the CMC + extract and nano‐extract, CMC + TBHQ treatment which had good quality to the end of the storage period. Ojagh, Rezaei, Razavi, and Hosseini (2010) also reported that by adding chitosan coating and cinnamon essential oil, the sensory analysis of preserved trout fillets was reduced compared to the control treatment. But overall, all treatments had sensory ratings approved by the evaluators.

## 4 CONCLUSION

The results of the study showed that CMC coating and sour tea extract have antioxidant properties and nanoencapsulation increased its antioxidant properties so that CMC coating and nano‐tea extract reduces the oxidative spoilage process in chicken nuggets. It significantly delayed and extended the shelf life of nuggets and was similar in all tests to the effect of TBHQ and in some cases even more effective. In general, the results of the study confirm the technology of using CMC and nano‐sour tea extract to enhance quality and shelf life of nugget. Therefore, the combination of CMC and nano‐sour tea extracts can provide the demand of consumers for chemical‐free meat products and healthy and safe food.
